# The Significance of COVID-19 Immunological Status in Severe Neurological Complications and Multiple Sclerosis—A Literature Review

**DOI:** 10.3390/ijms22115894

**Published:** 2021-05-31

**Authors:** Joanna Kulikowska, Agnieszka Kulczyńska-Przybik, Barbara Mroczko, Alina Kułakowska

**Affiliations:** 1 Department of Neurology, Medical University of Bialystok, 15-276 Bialystok, Poland; joanna.kulikowska@umb.edu.pl; 2 Department of Neurodegeneration Diagnostics, Medical University of Bialystok, 15-269 Bialystok, Poland; agnieszka.kulczynska-przybik@umb.edu.pl (A.K.-P.); mroczko@umb.edu.pl (B.M.)

**Keywords:** COVID-19, SARS-CoV-2, neuro-COVID, serology, antibodies, cerebrospinal fluid, multiple sclerosis

## Abstract

SARS-CoV-2/Coronavirus 2019 (COVID-19) is responsible for the pandemic, which started in December 2019. In addition to the typical respiratory symptoms, this virus also causes other severe complications, including neurological ones. In diagnostics, serological and polymerase chain reaction tests are useful not only in detecting past infections but can also predict the response to vaccination. It is now believed that an immune mechanism rather than direct viral neuroinvasion is responsible for neurological symptoms. For this reason, it is important to assess the presence of antibodies not only in the serum but also in the cerebrospinal fluid (CSF), especially in the case of neuro-COVID. A particular group of patients are people with multiple sclerosis (MS) whose disease-modifying drugs weaken the immune system and lead to an unpredictable serological response to SARS-CoV-2 infection. Based on available data, the article summarizes the current serological information concerning COVID-19 in CSF in patients with severe neurological complications and in those with MS.

## 1. Introduction

Coronavirus 2019 (COVID-19) is a newly emerging disease, which has caused a global pandemic as announced by the World Health Organization (WHO) in March 2020 [1]. Severe acute respiratory virus-2 (SARS-CoV-2), the virus responsible for COVID-19, has affected over 151 million people and contributed to over 3 million deaths [2]. SARS-CoV-2 is an enveloped positive-sense single-stranded RNA virus and is composed mainly of N (nucleocapsid), S (spike), M (membrane) and E (envelope) proteins [3,4]. Entrance into a host cell is induced by a connection between a spike protein and angiotensin-converting enzyme 2 (ACE2) receptor. The same mechanism was responsible for the SARS pandemic in 2002/2003 [5]. The incubation period of the disease is 2–14 days, and its main symptoms include fever, cough and shortness of breath [6]. A SARS infection can lead to pneumonia and acute respiratory distress syndrome that can result in death [7]. According to present data 99.6% cases are mild, and 0.4% are serious or critical [2]. The severe course mainly involves the elderly and patients with comorbidities [8,9]. However, recently, some new variants (such as B.1.1.7 [or VOC 202012/0], B.1.351 [or 501.V2], B.1.617) with many new mutations have emerged and are potentially more virulent and infectious, and more importantly, cause severe disease in young people in addition to the elderly [2,10]. Due to the presence of asymptomatic infections, the number of infected people remains underestimated [9]. For routine diagnostic processes, molecular tests, such as polymerase chain reaction in real time (qPCR), which indicates the acute phase of the disease, and serological tests that can detect specific antibodies are used [11]. The second type of test can determine whether the patient has had contact with the virus, determines its serological status and is used, among others, in epidemiological studies assessing the incidence of SARS-CoV-2 infection in the population [12]. A crucial issue is the body’s immune response to SARS-CoV-2 infection. During infection, an increase in the production of numerous proinflammatory cytokines, such as tumor necrosis alpha and interleukins-2 and -6 (TNF-α and IL-2 and -6, respectively) is observed [13]. A severe course of SARS-CoV-2 infection is undeniably connected with dysregulation of immune system and cytokine release syndrome [8]. Part of the immune response also involves the production of antibodies, mainly against S and N proteins, also known as neutralizing antibodies. The crucial role of these antibodies is to block virus entrance into host cell and activation of antibody-dependent cell cytotoxity (ADCC). As a result, the disease could be defeated, or the immune system’s overactivity could induce a cytokine storm [12]. In addition, patients showed a reduction in B- and T-lymphocytes and natural killer (NK) cells. Both the increase in inflammatory cytokines and the decrease in lymphocyte counts are associated with the severity of the disease [13]. Due to the fact that entry into the human cell is associated with the ACE2 receptor and these receptors also exist on neurons and glial cells, speculations about the neurotropism of SARS-CoV-2 began [14]. From the early stages of the pandemic, neurological symptoms have been described, of which the most common are anosmia, ageusia, headaches and dizziness (Table 1) [15]. In addition, the course of COVID-19 may be associated with much more severe neurological complications, such as encephalopathy, Guillain-Barre syndrome, meningitis, encephalitis and/or necrotizing hemorrhagic encephalopathy [15,16]. Moreover, the relationship between SARS-CoV-2 infection and acute cerebrovascular diseases, such as acute ischemic stroke, cerebral venous sinus thrombosis, cerebral hemorrhage and subarachnoid hemorrhage, were sought [15]. During studies on the neuroinvasiveness of SARS-CoV-2, the presence of anti-SARS-Cov-2 antibodies in the cerebrospinal fluid (CSF) and intrathecal synthesis were found; interestingly, in these cases, the PCR results from nasopharyngeal swabs remained negative [17]. Reports of possible cross-reactions with human proteins and formation of autoantibodies and as a consequence, development of autoimmune encephalitis have been published [18]. As previously mentioned, the severe course of COVID-19 is associated with the presence of comorbidities in people. It can be assumed that such comorbidities are neurological disorders, such as multiple sclerosis (MS). An interesting issue is the serological status of people with MS, especially those undergoing treatment with disease-modifying therapy. The current data suggest that even in the presence of a altered immune system, the risk of severe course and serological status are similar to those in the general population [19]. This review summarizes the current knowledge of severe neurological complications in COVID-19 and the serological status of those with neurological diseases.

## 2. Immune Response to SARS-CoV-2 Infection: Most Important Facts

A critical issue that is necessary for understanding the processes occurring during viral neuroinvasion is the immune response to SARS-CoV-2 infection. It is worth noting that 80% of the viral RNA is identical to that in SARS-CoV. Therefore, some mechanisms described in 2002/2003 can also be applied to the present pandemic [4,20]. After the virus enters the body, it is likely that any antigen can be presented by the major histocompatibility class 1 (MHCI), which leads to stimulation of both CD4 + and CD8 + T-cells [3,20]. As a consequence of many processes, the production of antibodies most often against the N protein and the receptor-binding domain (RBD) of the S protein occurs. Anti-S antibodies are called neutralizing antibodies [3]. It has been assumed that the early CD4 + and CD8 + response is protective, and if it is not inhibited after a while, the late response is excessive and destructive for the organism [21,22]. It is worth mentioning that the SARS-CoV-2 infection is divided into four phases, of which phase III is characterized by an abnormal inflammatory response, which is associated with systemic complications and extrapulmonary manifestation of the disease that led to phase IV infection, including acute respiratory distress syndrome (ARDS) and multi-organ failure [23,24]. In critically ill patients, a drastic decrease in T-lymphocytes has been noted [21,25]. Interestingly, this finding turned out to be inversely related to the amount of pro-inflammatory cytokines that were produced [21]. The recovery of the CD4 + and CD8 + populations was associated with their decline and improvement in the patient’s clinical condition [21]. This excessive inflammatory response is associated with a large number of pro-inflammatory cytokines, including interleukins-2, -6 and -7 (IL-2, -6 and -7,) macrophage inflammatory protein 1, monocyte chemoattractant protein 1, tumor necrosis factor alpha (TNFα) and colony-stimulating factor, and is called a cytokine storm [26]. A similar phenomenon has already been described in the case of infections with Ebstein-Barr virus as the so-called hemophagocytic lymphohistiocytosis with sudden and fatal hyperketonemia leading to the syndrome of multi-organ failure [20,27,28]. A cytokine outbreak leads to an influx of neutrophils to the lung parenchyma and, consequently, to excessive myelopoiesis [21]. The resulting immature neutrophils are believed to have immunosuppressive properties [21]. IL-6 and ferritin levels have proven to be associated with an increase in COVID-19 mortality [13]. This “viral sepsis-like syndrome” is the focal point when treating COVID-19 using a biological drug called Tocilizumab [23]. The drug is a monoclonal antibody directed against IL-6, which leads to an improvement in the lungs’ condition in 77% of patients [21,29]. In contrast to excessive inflammatory responses, CD8 + lymphocytes and interstitial macrophages in the lungs produce anti-inflammatory IL-10, which limits infection [21]. In summary, the body’s immune response may turn out to be either adequate to respond to the ongoing infection and result in disease control, or it may be excessive and fatal [26]. 

## 3. Serology in SARS-CoV-2 Infection

Currently, qPCR remains the gold standard for diagnosing an active SARS-CoV-2 infection. Despite the high sensitivity and specificity of the test, a risk of obtaining a false positive (sample contamination) or a false negative (incorrect storage of the collected material) results exists [2,30]. Serological tests are a valuable supplement to the diagnosis of SARS-CoV-2 as they enable retrospective detection of infection and remain irreplaceable in population studies. Moreover, they are cheaper, easier to make and the collected material is less complicated to store [3]. These types of tests are also invaluable in assessing the risk of infection in risk groups. To understand the mechanism of serological diagnostics, it is worth reviewing the structure of the SARS-CoV-2 virus. This virus consists of structural proteins, the most important of which in serological terms are the nucleocapsid protein (N) and the spike protein (S) as almost all serological methods detect the IgG and IgM produced against these proteins. The S protein is a homotrimer whose key element is the S1 subunit and the receptor binding domain (RBD) against which the neutralizing antibodies are being produced [3]. The S1 subunit has been shown to be the most specific for SARS-CoV-2, while the N protein is produced in the greatest amount [30,31]. It was shown that the antibodies against the N protein correspond more closely to the previous SARS-CoV-2 infection, while the antibodies against the S protein are part of the humoral response and have a greater neutralizing potential [32]. Coronaviruses are respiratory tract viruses, and because they come into contact with mucous membranes in the respiratory tract, they induce the formation of IgA antibodies, while at present, the diagnostics of IgM and IgG antibodies is of the greatest clinical importance [12]. Researchers disagree about the order in which seroconversion occurs in the SARS-CoV-2 infection. It has been reported that the typical order of production is IgM first after which IgG is absent [33]. However, other studies have shown that seroconversion occurs in a manner similar to other infections. A study of 173 patients with SARS-CoV-2 infections showed that conversion of total antibodies, IgM and IgG was 93.1%, 82.7% and 64.7%, respectively. The mean seroconversion times in this study were 11, 12 and 14 days, respectively [3]. Similarly, in a study on 214 patients, it was shown that the highest sensitivity involved detection of IgM antibodies to the S protein. In this study, the sensitivity in the IgM class was 77.1%, and IgG was 74.3%. For comparison, the sensitivity to the N protein was 68.2% in the IgM class and 70% in the IgG class [3]. The median detection of IgA of 5 days and IgG of 14 days were also compared. Comparing the IgM and IgA responses, the corresponding peak response was obtained on days 10–12 and 20–22 of the disease, respectively [34]. The IgA response turned out to be stronger and more durable than the response associated with IgM. A meta-analysis of 3856 confirmed cases showed that the positive index of IgM, IgG and their combined detection amounted to 61.2%, 58.8% and 62.1%, respectively. Furthermore, it turned out that IgM/IgG was detected in 19% of asymptomatic cases [35]. In addition to the enzyme-linked immunosorbent and chemiluminescent assay (ELISA and CLIA, respectively) methods that are most commonly used in serological diagnostics, it is worth mentioning the lateral flow immunoassay, which can detect total IgG or IgM even in 15 min [36]. The meta-analysis showed a sensitivity for these tests ranging from 72.2% to 100% and a specificity from 98.9% to 100% [36]. Another serological method is the magnetic chemiluminescence test, which is based on the double antibody sandwich method. This test demonstrates concurrent seroconversion for IgG and IgM. After 19 days of illness, 100% of patients had positive IgG. The antibodies reached a plateau after six days of seroconversion [37]. However, the ELISA test is still the most frequently used technique. The sensitivity, specificity and positive and negative predictive values for IgM were assessed as 87.5%, 100%, 100%, 95.2%, respectively, and for IgG, these values were 70.8%, 96.6%, 85%, 89% and 1%, respectively [3]. The mechanism of action of the antibodies produced by plasma cells occurs via prevention of virus entry into the cell by shielding the N and S proteins and activating antibody-dependent cytotoxicity. Interestingly, it was shown that patients with a more severe disease course had an earlier and higher antibody response. This phenomenon, called antibody-dependent enhancement (ADE), can be explained by the fact that anti-S antibodies inhibit viral entry into the cell, thereby enhancing the binding of the virus to B-cells and macrophages via the Fcγ-II receptor after which these cells are activated, and the cytokine storm intensifies [38]. Serological tests have their advantages, but their limitations should be kept into consideration. Researchers should be aware of the possibility of false-negative results in cases in which the patient has not yet developed antibodies. It is worth noting that despite the time of seroconversion determined in many studies, individual variability also exists. False positive results may be the result of a cross-reaction between other human coronaviruses. Determining antibodies also requires skillful interpretation of the selected test. Due to the above factors, serological tests remain additional tools in the diagnosis of the SARS-CoV-2 virus [35].

## 4. Cerebrospinal Fluid Serology: General Information

Substantial evidence in favor of the destructive effects of COVID-19 on the nervous system has been provided by studies conducted on post-mortem brain tissues collected from patients who had COVID-19. During an autopsy of people who died from COVID-19, the virus was detected in cortical neurons, and inflammatory response was observed in the brain tissue [39]. Moreover, the S1 protein has been shown to damage the endothelial cells in the brain by disrupting the blood-brain barrier (BBB) [40]. However, the exact mechanism action of COVID-19 virus on the central nervous system is still unknown. The most common neurological manifestations observed in patients with SARS-COV-2 infection are dizziness, headaches and confusion. In an extensive study by Saieght et al. involving 214 cases with confirmed SARS-CoV-2 infection, it was revealed that up to 36.4% of patients presented such neurological symptoms [41].

Most of the literature concerns serological diagnostic tests in the serum of patients with neuro-COVID diseases; few studies have been conducted in cerebrospinal fluid, and findings seem to be inconsistent (Table 2 and Table 3). Before discussing the reports on serology, in particular neuro-COVID diseases, it is worth becoming acquainted with the general issues concerning antibodies in the cerebrospinal fluid and its biochemical features in case of neurological symptoms. It would be interesting for researchers to determine whether this unexceptional virus neurotropism is detected in cerebrospinal fluid (CSF) and whether a specific CSF biomarker exists for neuroinvasion. In the beginning, it is worth noting that serology turned out to be much more useful for the assessment of neuroinvasion than did the PCR test. One of the first studies concerning neurological patients revealed that two patients with ischemic stroke and confirmed COVID-19 based on nasopharyngeal swabs had negative PCR results from the cerebrospinal fluid [41]. In the next study, thirty patients with neurological symptoms were examined, and none of them showed positive PCR results from CSF samples [42]. Similar findings were obtained from one of the largest retrospective studies. In 555 CSF samples from patients with genetically confirmed COVID-19, all but two samples had negative PCR results. Interestingly, two positive samples were collected post-mortem [43]. On the other hand, inconsistent data are also found in the literature. A study by Moriguchi et al. confirmed direct invasion by COVID-19 into the nervous system. In the first described case of meningitis, the authors observed negative PCR from the swab and positive from the CSF [16]. These findings agreed with another study in which subjects suspected of demyelination associated with SARS-COV-2 infection also had positive PCR results from CSF samples [44]. The positive CSF PCR seems to be related to more serious neurological manifestations. So far, no large studies have been conducted to assess the presence of anti-SARS-CoV-2 antibodies in the CSF. However, findings from smaller studies indicate a substantial role for SARS-COV-2 antibodies in CSF. Eight patients hospitalized in the intensive care unit (ICU) were examined, and each of them had antibodies against SARS-COV-2 virus in the CSF as determined using the ELISA method (Euroimmun, Lübeck, Germany). However, none of the detected autoantibodies were characteristic for autoimmune encephalitis (anti-N-methyl-D-aspartate receptors, NMDAr, alpha-amino-3-hydroxy-5-methyl-isoxale-type glutamate, gamma aminobutyric acid, contact-associated protein-like 2, dipeptidyl-peptidase-like protein, leucine-rich and glioma-inactivated 1 receptors (AMPAr, GABAbr, CASPR2, DPPX and LGI1, respectively). Similarly, CSF PCR results were negative in all patients. Interestingly, in one patient, intrathecal antibody synthesis was detected, and in three subjects, disruption of the BBB was detected. Additionally, three patients were positive for the 14-3-3 protein, which indicated an ongoing neurodegenerative process [17]. In the case report of two patients with neurological symptoms, antibodies against SARS-CoV-2 were detected in the CSF. The leakage rates were 1.08% and 3.12%, which indicate that the presence of these antibodies in the cerebrospinal fluid was due to leakage rather than intrathecal production. Additionally, the presence of the viral proteins N, S1 and S2 was detected. The samples were strongly positive for N and weaker but also positive for the S1 and S2 proteins [45]. A growing body of evidence indicates the possibility of autoantibody formation during the course of SARS-CoV-2 infection, which is very interesting due to the possible mechanism explaining the course of neuroinvasion. Franke et al. assessed serum levels of autoantibodies in 11 critically ill patients with confirmed COVID-19 who presented new neurological symptoms, such as myoclonus, seizures, dystonias and oculomotor disturbances. Interestingly, all tested patients showed anti-Yo receptor or anti-NMDA receptor antibodies in addition to “various specific, undefined epitopes, resembling the binding of brain tissue observed with some human SARS-CoV-2 monoclonal antibodies” [46]. A few literature studies suggest that neurological diseases may develop as a complication following SARS-CoV-2 infectious. For example, a Spanish group of researchers reported a patient with asymptomatic SARS-CoV-2 infection and hypoesthesia in the limbs and perineum. The radiographic image corresponded to acute transverse myelitis, and CSF tests showed bands corresponding to IgM anti-GD2 and GD3 antibodies [47]. A negative PCR result and the presence of antibodies or autoantibodies strongly suggest that the indirect mechanism of SARS-CoV-2 is via an attack on the nervous system. Some researchers suggest that secondary damage to the nervous system cells may be caused by the phenomenon of immune mimicry between viral proteins and human antigens as described, for example, in the case of encephalitis after infection with the herpes simplex virus. The phenomenon of molecular mimicry consists in exterminating the structural similarity between antigens of microorganisms and human ones. As a consequence, in cases in which an infection occurs, the resulting antibodies will attack not only the microorganism but also its own tissue antigens [48,49]. Many other viruses, such as other coronaviruses, adenoviruses and enteroviruses, are also suspected of inducing autoimmune processes [50]. Perhaps the neuro-COVID syndrome will prove to be similar to the lethargic encephalitis that occurred during the influenza pandemic of 1918 [51]. The hypothesis of the autoimmune mechanism of neurological symptoms may be confirmed by studies on autopsy cases in which pathological changes, mainly in the brainstem and cerebellum corresponding to the picture of autoimmune encephalitis, were described. The presence of the virus was detected in half of the tested preparations; however, it did not correlate with the severity of the brain damage, which further supports the immune mechanism of neuroinvasion [52]. A literature review also allows general conclusions to be drawn about the changes in the CSF study. Pleocytosis is very common and usually presents with increased lymphocytes and possible elevated levels of protein. An increase in the level of albumin indicates damage to the BBB. Moreover, in some patients, oligoclonal bands were found [48,53]. Additionally, reports in the literature can be found in which levels of pro-inflammatory cytokines (IL-1 and monocyte chemoattractant protein [MCP]-1) in CSF were determined, and it was found that they remained elevated [54]. Encephalopathy and other neurological symptoms are seen in many seriously ill patients, possibly due to several mechanisms: (1) direct viral neuroinvasion may occur, which is explained by the presence of ACE2 receptors in cells of the nervous system, (2) neurological symptoms occur as a result of systemic biochemical and metabolic complications and (3) the BBB can be damaged by an excessive inflammatory response and multiple mediators. In the light of the cited studies, the latter hypothesis seems to be the most justified as it explains the negative PCR in most studies and the presence of SARS-CoV-2 antibodies, autoantibodies and abnormalities in the general CSF test [17].

## 5. Serology in Severe Neurological Manifestations of COVID-19

### 5.1. Meningoencephalitis and Autoimmune Encephalitis

Meningoencephalitis has a wide possible etiology. Viruses are known causes of this disease; they are responsible for often varied clinical picture and characteristic changes when examining the CSF. The SARS-CoV-2 virus was suspected of causing meningitis and meningoencephalitis during the first months of the pandemic. In the first described case, PCR from the nasopharynx was negative with a positive result from the CSF. At that time, the CSF was not tested for the presence of antibodies and autoantibodies [16]. One of the largest studies to date that included patients with meningitis was a meta-analysis of 54 cases of meningitis in patients with COVID-19. Interestingly, the virus was detected in the CSF of three patients. Additionally, in the analyzed CSF results, the following laboratory tests turned out to be statistically significant: (1) total protein (n = 18; *p* = 0.004), (2) lymphocytes (n = 6; *p* = 0.009) and (3) IgG (n = 5; *p* < 0.0001). The described studies showed a statistically significant reduction in IgG levels, which could be explained by the hypoglobulinemia resulting from lymphopenia [55,56]. Contradictory results are presented in a study by the scientists from Wuhan, who found meningeal symptoms in a patient with SARS-CoV-2 and demonstrated a positive PCR swab result but did not detect any antibodies in the cerebrospinal fluid despite a diagnosis of COVID-19-related meningitis [57]. Anti-N-methyl-D-aspartate (NMDA) autoantibody encephalitis is a neurological and psychiatric symptomatic disorder, often on the psychotic spectrum. During the pandemic, reports concerning the presence of autoantibodies in the CSF of patients with encephalitis after COVID-19 infection have been published. For example, in a 23-year-old man from Ecuador with COVID-19 who developed psychotic symptoms had anti-NMDA antibodies in the CSF and was diagnosed with autoimmune encephalitis [58]. Likewise, in a 50-year-old male with COVID-19 symptoms, development of refractor epilepticus status was suspected because anti-NMDA antibodies in the CSF in addition to pleocytosis and oligoclonal bands were detected [59]. 

### 5.2. Acute Myelitis 

Acute myelitis is a disease with various possible etiologies. It is often the first symptom of other neurological diseases, such as MS [60]. Moreover, it is also often associated with a previous viral or bacterial infection that triggered autoimmune mechanisms [61]. During the SARS-CoV-2 pandemic, myelitis in patients with COVD-19 has been reported. In a study, PCR from CSF samples, antibodies and autoantibodies were found. The most common acute myelitis-associated symptoms are sensory disturbances, paresthesia, paresis and spinal pain. The radiographic images most often show hyperintense signals on T2 images, characterized by longitudinal myelitis [62]. In study of COVID myelitis cases, it was found that the most common changes in the cerebrospinal fluid were lymphocytosis and elevated protein levels. Oligoclonal bands were usually negative. The CSF PCR test was negative in each analyzed case [47]. An interesting case was presented by a 60-year-old woman with transverse myelitis with positive IgM anti-GD2 and IgM anti-GD3 antibodies [47]. One case report suggests the possibility of a lupus anticoagulant in acute myelitis accompanying the COVID-19 infection. Literature data show COVID-19 and lupus anticoagulant coexistence in 45% to 87% of patients, especially those hospitalized in the intensive care unit (ICU). No studies assessing the relationship between lupus anticoagulant and neurological complications have been published [63]. A review of the literature indicates more cases of myelitis (about 15 studies so far) while the analysis of the tests performed allows us to state that the determination of antibodies against SARS-CoV-2 in CSF is not a routine test, which does not allow clear conclusions about the serological status of these patients to be drawn.

### 5.3. Guillain-Barré Syndrome

Guillain-Barré syndrome (GBS) and its variants, such as Miller-Fisher syndrome, is an acute autoimmune neuropathy that can lead to ARDS and consequently, to death. The most frequently reported symptoms of GBS include symmetrical limb paresis, cranial nerves palsy and sensory disturbances. It is recognized that the disease is related to a recent bacterial or viral infection [64]. The phenomenon of molecular mimicry is the mechanism responsible for GBS pathogenesis. The data show a cross-reaction between pathogenic antigens and com peripheral nerve components, mainly gangliosides. The antibodies anti-gangliosides, GM1, GM2, GD1a, GD1b and GQ1b, are included in the panel of the most frequently measured antibodies. The most common antibody in GBS is anti-GM1, while anti-GQ1b is associated with the Miller–Fisher variant [65,66]. During the COVID-19 pandemic, an increase in the number of GBS cases was observed, and interestingly, the average age of patients in these pandemic-associated cases was also higher than in GBS cases before COVID-19 [67]. A few review articles about the relationship between GBS and COVID-19 have appeared in the literature so far. In the most recent study, they reported that of 36 cases in which the autoantibody panel was evaluated, 31 were negative and five were positive. Interestingly, no case of anti-GM1 antibodies has been reported in classical GBS, and no case of anti-GQ1b antibodies in Miller–Fisher variant [65]. As shown above, the vast majority of cases remained negative for the presence of anti-ganglia antibodies that are characteristic of pre-pandemic GBS in up to 88% of patients [66]. In the analyzed cases, the typical GBS treatment (an infusion of intravenous immunoglobulins) was used with great effectiveness [65]. This finding suggests that GBS after COVID-19 may have an autoimmune basis. Based on this information, it is suspected that the SARS-CoV-2 virus induces an autoimmune response through different autoantigens than before [65]. One theory states that the presence of anti-ganglioside antibodies is related to the axonal subtype of GBS, while the lack of these antibodies suggests a demyelinating effect [68]. This process gives rise to the assumption that the SARS-CoV-2 virus may cause demyelination in the nervous system. In the case reports published so far, routine determination of antibodies against SARS-CoV-2 has not been done. Therefore, the serological status of these patients is unknown. Positive CSF PCR was not demonstrated in any of the cases described in these papers [65,68]. An interesting case was presented in a study describing a patient who returned to the hospital after COVID-19 with neurological symptoms, including dizziness, eye movement disorders and disorientation. Anti-GD-1 antibodies were found in the serum, and Bickerstaff encephalitis was diagnosed [69,70].

## 6. Serology in Multiple Sclerosis in the Course of COVID-19

Comorbidities are an acknowledged risk factor of severe COVID-19. MS has been considered such a disease since the beginning of the pandemic. MS is an autoimmune disease in which demyelination occurs in the central nervous system as a result of autoantibody formation in response to myelin proteins. Concern over the severe course of COVID-19 is caused not only by the disease itself, in which the immune system remains altered, but also by disease-modifying treatments (DMTs). These drugs cause various types of immunosuppression and can affect both the course of the infection and the serological response after infection [71]. Interestingly, viral infections, including coronaviruses, can be a pathogenetic factor in multiple sclerosis. In addition, intrathecal synthesis of anti-HCoV antibodies against coronaviruses has been reported in MS patients [14,72]. Currently, various disease-modifying drugs, which have different mechanisms and differently influence the serological response after an illness, are used in the treatment of MS (Table 4). To date, reports of some of these drugs affecting the presence of seroconversion following SAS-CoV-2 infection can be found [71]. Cladribine is a drug that restores the immune system and has proven long-term efficacy. Concerns about this therapy are related to the risk of severe infections due to possible lymphocyte depletion [73]. The literature describes a case of a 35-year-old woman with MS who underwent two 5-day cycles of cladribine treatment a year ago and developed a mild SARS-CoV-2 infection. Antibodies to SARS-CoV-2 were positive three months after the disease (no antibody class or method of determination was given) [74]. Another patient with a short disease duration, who was using cladribine from January 2020 and had mild lymphopenia (0.9 × 10^9^/L) did not develop a serological response even after four months after contracting a SARS-CoV-2 infection. A 40-year-old patient described in the same article who had a long history of the disease and was treated with cladribine in February and March 2020 achieved seroconversion four months after contracting COVID-19 [75]. Contradictory findings were obtained by Gelibter et al., who reported a patient also with mild COVID-19 but who did not seroconvert IgM and IgG. The lymphocyte count was then 730/µL [22,76]. Anti-CD20 monoclonal antibodies, such as rituximab and ocrelizumab, are increasingly used drugs, especially in the forms of primary progressive MS. A reduction in the immunoglobulin production, mainly IgG, has been documented. At the beginning of the pandemic, the question arose as to whether people taking anti-CD-20 monoclonal antibodies could be at risk of more severe COVID-19 infections and whether they could develop normal immunity after exposure or vaccination [77]. The literature presents a case of a 65-year-old patient who had mild COVID-19 and in whom positive IgA antibodies to SARS-CoV-2 were detected 10 weeks after the infection, while IgG antibodies remained negative [78]. IgA antibodies are associated with the immune system of the mucous membranes, and the detection of IgA antibodies to SARS-CoV-2 in patients treated with ocrelizumab may indicate a lower effect of this drug on mucosa-associated lymphoid tissue (MALT) compared to other components of the immune system [79]. In another study, five patients treated with anti-CD-20 therapy had negative IgG antibodies. In the same study, another six patients treated with other disease-modifying drugs (teriflunomide, glatiramer acetate, dimethyl fumarate, natalizumab) showed IgG seroconversion. In the above-mentioned study, the minimum time between COVID-19 and antibody testing was 23 days [80]. The literature also includes an example of two patients with relapsing–remitting MS that was treated with ocrelizumab; one patient was a 39-year-old woman and the other was a 42-year-old male after ambulatory-treated COVID-19. Neither of them showed seroconversion in the IgG class. Antibody levels were tested in the female at week 6 and 12 after infection, and in the male at week 7 and 9 post-infection [81]. Similar results were obtained in a 48-year-old woman treated with ocrelizumab, in whom IgG anti-SARS-CoV-2 antibodies were not detected three months after recovering from COVID-19 despite double testing within a few days [82]. In one study, 84 out of 93 MS patients, whether treated with disease-modifying therapy (DMT) or not, were tested by PCR, serological tests, or both. In 79 patients, serological tests were performed three months after obtaining a positive PCR test. In this study, a serological response was achieved in 17.6% of patients treated with anti-CD-20, 48.8% of those treated with other DMTs and 68.4% of untreated patients [83]. Another drug that binds to CD-20 epitopes is ofatumumab, which is also used in multiple sclerosis treatment [84]. One case report describes a patient suffering from MS who was treated with ofatumumab and showed the presence of IgG and IgM antibodies against the spike protein three months after contracting COVID-19 [85]. Other medications used to treat MS include fingolimod and teriflunomide. Fingolimod is a sphingosine 1-phosphate type 1 receptor modulator that stops the migration of T-lymphocytes from the lymph nodes to the central nervous system, thus limiting the autoimmune process [86]. Teriflunomide inhibits *de novo* pyrimidine synthesis by blocking the enzyme dihydrouridine dehydrogenase [86]. In two post-COVID patients who had been treated with fingolimod or teriflunomide, the serum levels of IgG antibodies against the S1 or N protein of SARS-CoV-2 virus on days 7, 21, 28 and 35 from the time of coronavirus infection diagnosis were examined. The patient treated with fingolimod achieved a limited serological response only against the N protein, which was defined as being slightly positive at day 35, whereas in a patient treated with teriflunomide detected anti-N and -S antibodies on day 21 after diagnosis of SARS-CoV-2 infection [87]. Similar differences in the immune response of these substances have been shown after vaccination with seasonal influenza [88].

## 7. Discussion

SARS-CoV-2, which is responsible for the pandemic that started in December 2019, is the cause of significant medical problems. Within a year, this new virus was isolated, its structure was described, both the pathogenesis of the new disease and associated symptoms were characterized, diagnostic methods were introduced, a treatment was proposed and most importantly, an effective vaccine was developed. Currently, the diagnostic standard is the PCR test, which detects the genetic material of the virus [1]. However, serological diagnostics also provides very important information, not only about the history of the disease, but also about the population immunity and the potential capability of an immune response to the administered vaccine. Serological tests are based on antibody detection against SARS-CoV-2 virus antigens, most often against the N and S1 proteins, which are characterized by the highest immunogenicity compared to other viral proteins [89]. The severe course of the disease is associated with the phenomenon of a cytokine storm in which excessive and abnormal immune responses to ongoing infection occur [4]. Based on the available data, this article focuses on characterizing the serological statuses of patients who present with neurological complications after SARS-CoV-2 infection and patients with MS who have had COVID-19. Antibodies to the SARS-CoV-2 virus, apart from the blood serum, can also be identified in the cerebrospinal fluid [17]. The available data show that despite frequent neurological complications, antibody detection is not yet used as a standard procedure. In such situations, a PCR test is performed more frequently although the current data indicate that the mechanism responsible for the development of neuro-COVID is an immune mechanism rather than direct viral neuroinvasion [17]. This difference is indicated by a negative CSF PCR test in almost all of the described cases with a single exception [16]. On the other hand, just a few studies have been conducted to assess the presence of anti-SARS-CoV-2 antibodies in the CSF. The available data indicate the presence of such antibodies, especially in patients hospitalized in the ICU [17]. The presence of anti-SARS-CoV-2 antibodies in the CSF can be explained by damage to the BBB caused by a cytokine storm rather than by intrathecal synthesis [90]. Cases of autoimmune encephalitis during or after COVID-19 can be found in the literature. Interestingly, despite the presence of clinical and radiological symptoms, in most of the described patients’ autoantibodies typical for this disease could not be identified [17,46]. This situation is similar to that of GBS and acute myelitis, which is the reason for concluding that over the course of COVID-19, the immune system is stimulated with other than the previously identified autoantigens of the nervous system, which have not been identified so far [64]. A positive 14-3-3 protein was detected in samples from a few patients, which indicates a possible initiation of the neurodegenerative process due to SARS-CoV-2 infection [17]. More data on serology related to COVID-19 can be found on patients with multiple sclerosis. From the beginning of the pandemic, it has aroused the interest of researchers whether MS patients are at risk of severe COVID-19 [31]. The available data tend to negate these concerns [91]. Many people with MS are treated with DMTs, which often cause immunosuppression and can interfere with the serological responses to both past infection and vaccination [92]. Many DMTs, such as anti-CD20 drugs, present great concern with respect to immunosuppression.

Interestingly, the production of antibodies, mainly in the IgG class, against the SARS-CoV-2 virus in patients taking these medications turned out to be the weakest [81,83]. In one study, the researchers determined the presence of positive IgA antibodies, which could be explained by the fact that ocrelizumab has a lower effect on MALT tissues than did other similar drugs [93]. Conflicting data about the serological responses can be found with cladribine. Out of four analyzed cases, three patients seroconverted, and one did not [74,75,76]. For other DMTs (such as: glatiramer acetate, dimethyl fumarate, natalizumab, teriflunomide) IgG seroconversion has been described [80]. Only one patient treated with fingolimod was found who was given a serology test after COVID-19 infection. This patient seroconverted slightly positive against the N protein, whereas antibodies against the S protein were negative [87]. Based on this case, it is difficult to draw conclusions regarding the entire group of patients treated with these drugs.

## 8. Conclusions

In summary, serological tests are an integral part of COVID-19 diagnostics and provide a great deal of valuable information. Up to 36% of patients with SARS-CoV-2 infection show neurological symptoms. Furthermore, with the progression of the pandemic, the range of neurological symptoms is becoming more extensive and also includes serious complications, such as autoimmune encephalitis or GBS [7,41]. However, it is not yet well understood whether these symptoms are due to direct damage to the nervous system by the virus or via the indirect consequences of an infection. The available data indicate immune pathogenesis rather than direct viral neuroinvasion [14]. Therefore, it can be concluded that in the diagnosis of such patients, antibodies to COVID-19, including those in the CSF, should be evaluated in order to understand better biological mechanisms underlying neurological manifestation of SARS-CoV-2 infection and also to confirm viral etiology. Unfortunately, this diagnostic procedure is not standard today. It is hoped that such a procedure will be included in future guidelines for neuro-COVID diagnostics. More data are available for multiple sclerosis, especially for patients treated with disease-modifying drugs. The capability of their immune systems to produce antibodies is particularly important in terms of administering preventive vaccinations in this group of patients. However, still a lack of data on the serological status of the entire group of patients with multiple sclerosis still exists. Moreover, further studies are needed to assess post-infection immunity in the population of neurological patients. As we gain more knowledge about the different ways in which COVID-19 can destroy the human body, understanding the neurological symptoms will be pivotal in helping people recover from this severe viral infection.

## Figures and Tables

**Table 1:** Neurological symptoms and possible complications related to severe acute respiratory coronavirus -2 (SAR-CoV-2) infection.

Coronavirus-2019 (COVID-19) and Neurological Disturbances		
Neurological Symptoms	Severe Neurological Complications	COVID-19 Complicates Course of Neurological Diseases
Headaches Dizziness Seizures Anosmia/hyposmia Ageusia/hypogeusia Hypoesthesia Paresis and paralysis Disturbances of consciousness Urination disorders	Guillain-Barre Syndrome and Miller-Fisher Syndrome Acute Transverse Myelitis Encephalopathy Demyelination Encephalitis/meningoencephalitis Autoimmune encephalitis Necrotizing hemorrhagic Encephalopathy Ischemic stroke Cerebral hemorrhage Cerebral venous sinus thrombosis Subarachnoid hemorrhage	Multiple sclerosis Neuromyelitis optica spectrum disorders Epilepsy Amyotrophic lateral sclerosis, Parkinson disease Dementia

**Table 2:** Serology and polymerase chain reaction (PCR) in the cerebrospinal fluid (CSF) of patients with neurological manifestation during SARS-CoV-2 infection.

N	Neurological Complication	PCR	PCR in CSF	Serology	Antibody Class	Time after Infection	Metodology	Ref.
2 patients	Ischemic stroke	+	-	Not given				[7]
30 patients	Guillain-Barre syndrome, encephalophaty, seizures, ischaemic stroke and others	+	−	Not given				[8]
555 patients	Headaches, meningitis or no neurological symptoms	+	+ (in 2/555)	Not given				[9]
Case report	Meningitis	−	+	Not given				[10]
Case report	Demyelination	−	+	Not given				[11]
8 ICU	Encephalopathy	+	Not given	+	IgG	During hospitalization	ELISA	[1]
2 patients	Encephalopathy	+ +	Not given	+ +	Not given	>16 days During hospitalization	ELISA	[12]
11 ICU patients	Myoclonus, seizures, dystonias, oculomotor disturbances	+	Not given	anti-Yo receptoranti-NMDA receptor antibodies	Not given	During hospitalization	Cell-based assays and indirect immune fluorescence	[13]
Case report	Hypoaesthesia in the limbs and perineum	+	−	anti-GD2 and GD3 +	IgM	During hospitalization	not given	[14]

Abbreviation: Intensive care unit (ICU), PCR: polymerase chain reaction, ELISA: enzyme-linked polymerase chain reaction.

**Table 3:** Serology in the severe neurological manifestation of Coronavirus 2019 (COVID-19).

	N	PCR Swab	PCR in CSF	Antibodies	References
Meningoencephalitis and autoimmune encephalitis	Case report	−	+	Not tested	[10]
	54 patients	+	+ (in 3 patients)	Not tested	[15]
	2 patients	+	Not tested	Anti-NMDA	[16,17]
Acute myelitis	10 Patients	+	− (in all)	Not tested	[14]
	Case report	+		IgM ant GD2/GD3	[14]
	Case report	+	Not tested	Lupus anticoagulant	[18]
Guillain-Barré syndrome	Case report	+	Not tested	Anti-GD1	[19]
	83 patients	+	Not given	− in 31 out 36 tested + in 5 out of 36 tested (ani-G1b, anti- asialo GM1, anti-GD1a, anti-GM2)	[20]

**Table 4:** Multiple sclerosis: serology results post-COVID-19.

Disease-Modifying Therapy	Group of Patients	Serological Response in Plasma	Antibody Class	Time after SARS-CoV-2 Infection	Ref.
Cladribine	Case report	+	Not given	3 months	[23]
	Case report	+	Not given	4 months	[24]
	Case report	−	Not given	4 months	[24]
	Case report	− +/− (?)	IgM IgG	2 months	[22]
Anti-CD-20	Case report	+ −	IgA IgG	10 weeks	[25]
	Case report	+ +/− (?)	IgM IgG	3 months	[26]
	Case report	−	IgG	3 months	[27]
	2 patients	−	IgG	6–12 weeks	[28]
	5 patients	−	IgG	23–64 days	[29]
Glatiramer	2 patients	+	IgG	51–54 days	[29]
Dimethyl fumarate	2 patients	+	IgG	40–71 days	[29]
Natalizumab	1 patients	+	IgG	68 days	[29]
Teriflunomid	2 patients	+	IgG	66 days	[29]
	Case report	+ +/− (?)	IgG anti- S1 IgG anti-N	21 days	[30]
Fingolimod	Case report	− +	IgG anti S1 IgG anti N	35 days	[30]

## Data Availability

No new data were created or analyzed in this study. Data sharing is not applicable to this article.

## References

[1] Zhu N., Zhang D., Wang W., Li X., Yang B., Song J., Zhao X., Huang B., Shi W., Lu R. (2020). A Novel Coronavirus from Patients with Pneumonia in China, 2019. N. Engl. J. Med..

[2] Coronavirus Update (Live): 102,061,812 Cases and 2,201,477 Deaths from COVID-19 Virus Pandemic-Worldometer. https://www.worldometers.info/coronavirus/.

[3] Assadiasl S., Fatahi Y., Zavvar M., Nicknam M.H. (2020). COVID-19: Significance of antibodies. Hum. Antibodies.

[4] Li G., Fan Y., Lai Y., Han T., Li Z., Zhou P., Pan P., Wang W., Hu D., Liu X. (2020). Coronavirus infections and immune responses. J. Med. Virol..

[5] Graham R.L., Donaldson E.F., Baric R.S. (2013). A decade after SARS: Strategies for controlling emerging coronaviruses. Nat. Rev. Microbiol..

[6] Alhazzani W., Møller M.H., Arabi Y.M., Loeb M., Gong M.N., Fan E. (2020). Clinical Characteristics of Coronavirus Disease 2019 in China. N. Engl. J. Med..

[7] Huang C., Wang Y., Li X., Ren L., Zhao J., Hu Y., Zhang L., Fan G., Xu J., Gu X. (2020). Clinical features of patients infected with 2019 novel coronavirus in Wuhan, China. Lancet.

[8] Bhatt P.J., Shiau S., Brunetti L., Xie Y., Solanki K., Khalid S., Mohayya S., Au P.H., Pham C., Uprety P. (2020). Risk Factors and Outcomes of Hospitalized Patients with Severe Coronavirus Disease 2019 (COVID-19) and Secondary Bloodstream Infections: A Multicenter Case-Control Study. Clin. Infect Dis..

[9] Wu C., Chen X., Cai Y., Zhou X., Xu S., Huang H., Zhang L., Zhou X., Du C., Zhang Y. (2020). Risk Factors Associated with Acute Respiratory Distress Syndrome and Death in Patients with Coronavirus Disease 2019 Pneumonia in Wuhan, China. JAMA Intern Med..

[10] Zhang L., Jackson C.B., Mou H., Ojha A., Rangarajan E.S., Izard T., Farzan M., Choe H. (2020). SARS-CoV-2 spike-protein D614G mutation increases virion spike density and infectivity. Nat. Commun..

[11] (2020). Diagnosis and treatment plan of Corona Virus Disease 2019 (tentative sixth edition). Glob. Health J..

[12] Zhao J., Yuan Q., Wang H., Liu W., Liao X., Su Y., Wang X., Yuan J., Li T., Li J. (2020). Antibody responses to SARS-CoV-2 in patients of novel coronavirus disease 2019. Clin. Infect. Dis..

[13] Zhang B., Zhou X., Zhu C., Song Y., Feng F., Qiu Y., Feng J., Jia Q., Song Q., Zhu B. (2020). Dysregulation of immune response in patients with COVID-19 in Wuhan, China. Clin. Infect. Dis..

[14] Yachou Y., El Idrissi A., Belapasov V., Ait Benali S. (2020). Neuroinvasion, neurotropic, and neuroinflammatory events of SARS-CoV-2: Understanding the neurological manifestations in COVID-19 patients. Neurol. Sci..

[15] Zhou Z., Kang H., Li S., Zhao X. (2020). Understanding the neurotropic characteristics of SARS-CoV-2: From neurological manifestations of COVID-19 to potential neurotropic mechanisms. J. Neurol..

[16] Moriguchi T., Harii N., Goto J., Harada D., Sugawara H., Takamino J., Ueno M., Sakata H., Kondo K., Myose N. (2020). A first case of meningitis/encephalitis associated with SARS-Coronavirus-2. Int. J. Infect. Dis..

[17] Alexopoulos H., Magira E., Bitzogli K., Kafasi N., Vlachoyiannopoulos P., Tzioufas A., Kotanidou A., Dalakas M.C. (2020). Anti-SARS-CoV-2 antibodies in the CSF, blood-brain barrier dysfunction, and neurological outcome Studies in 8 stuporous and comatose patients. Neurol. Neuroimmunol. Neuroinflamm..

[18] Kreye J., Reincke S.M., Prüss H. (2020). Do cross-reactive antibodies cause neuropathology in COVID-19?. Nat. Rev. Immunol..

[19] Capasso N., Palladino R., Montella E., Pennino F., Lanzillo R., Carotenuto A., Petracca M., Iodice R., Iovino A., Aruta F. (2020). Prevalence of SARS-CoV-2 Antibodies in Multiple Sclerosis: The Hidden Part of the Iceberg. J. Clin. Med..

[20] Chowdhury M.A., Hossain N., Kashem M.A., Shahid M.A., Alam A. (2020). Immune response in COVID-19: A review. J. Infect. Public Health.

[21] Melenotte C., Silvin A., Goubet A.G., Lahmar I., Dubuisson A., Zumla A., Raoult D., Merad M., Gachot B., Hénon C. (2020). Immune responses during COVID-19 infection. Oncoimmunology.

[22] Zarrilli G., Angerilli V., Businello G., Sbaraglia M., Traverso G., Fortarezza F., Rizzo S., Gaspari M.D., Basso C., Calabrese F. (2021). The Immunopathological and Histological Landscape of COVID-19-Mediated Lung Injury. Int. J. Mol. Sci..

[23] Flisiak R., Parczewski M., Horban A., Jaroszewicz J., Kozielewicz D., Pawłowska M., Piekarska A., Simon K., Tomasiewicz K., Zarębska-Michaluk D. (2020). Management of SARS-CoV-2 infection: Recommendations of the polish association of epidemiologists and infectiologists. Annex no. 2 as of October 13, 2020. Polish Arch. Intern Med..

[24] Wong N.A., Saier M.H. (2021). The SARS-Coronavirus Infection Cycle: A Survey of Viral Membrane Proteins, Their Functional Interactions and Pathogenesis. Int. J. Mol. Sci..

[25] Tan L., Wang Q., Zhang D., Ding J., Huang Q., Tang Y.Q., Wang Q., Miao H. (2020). Lymphopenia predicts disease severity of COVID-19: A descriptive and predictive study. Signal Transduct. Target Ther..

[26] Mehta P., McAuley D.F., Brown M., Sanchez E., Tattersall R.S., Manson J.J. (2020). COVID-19: Consider cytokine storm syndromes and immunosuppression. Lancet.

[27] Maggi E., Walter Canonica G., Moretta L. (2020). COVID-19: Unanswered questions on immune response and pathogenesis. J. Allergy Clin. Immunol..

[28] WIESŁAW WIKTOR JĘDRZEJCZAK: Limfohistiocytoza Hemofagocytarna–Rzadko Rozpoznawany Uleczalny Stan Bezpośredniego Zagrożenia Życia Występujący Również u Dorosłych. http://pthit.pl/Acta_Haematologica_Polonica,Choroby_histiocytow_Cytokiny_Wirus_Epsteina_Barr,406.html.

[29] Tomasiewicz K., Piekarska A., Stempkowska-Rejek J., Serafińska S., Gawkowska A., Parczewski M., Niścigorska-Olsen J., Łapiński T.W., Zarębska-Michaluk D., Kowalska J.D. (2020). Tocilizumab for patients with severe COVID-19: A retrospective, multi-centre study. Expert Rev. Anti Infect. Ther..

[30] Bastos M.L., Tavaziva G., Abidi S.K., Campbell J.R., Haraoui L.P., Johnston J.C., Lan Z., Law S., MacLean E., Trajman A. (2020). Diagnostic accuracy of serological tests for covid-19: Systematic review and meta-analysis. BMJ.

[31] Bond K., Williams E., Howden B.P., Williamson D.A. (2020). Serological tests for COVID-19. Med. J. Aust..

[32] McAndrews K.M., Dowlatshahi D.P., Dai J., Becker L.M., Hensel J., Snowden L.M., Leveille J.M., Brunner M.R., Holden K.W., Hopkins N.S. (2020). Heterogeneous antibodies against SARS-CoV-2 spike receptor binding domain and nucleocapsid with implications for COVID-19 immunity. JCI Insight.

[33] Li K., Huang B., Wu M., Zhong A., Li L., Cai Y., Wang Z., Wu L., Zhu M., Li J. (2020). Dynamic changes in anti-SARS-CoV-2 antibodies during SARS-CoV-2 infection and recovery from COVID-19. Nat. Commun..

[34] Padoan A., Sciacovelli L., Basso D., Negrini D., Zuin S., Cosma C., Faggian D., Matricardi P., Plebani M. (2020). IgA-Ab response to spike glycoprotein of SARS-CoV-2 in patients with COVID-19: A longitudinal study. Clin. Chim. Acta.

[35] Guo C.C., Mi J.Q., Nie H. (2020). Seropositivity rate and diagnostic accuracy of serological tests in 2019-nCoV cases: A pooled analysis of individual studies. Eur. Rev. Med. Pharmacol. Sci..

[36] Diagnostic Performance of COVID-19 Serology Assays-PubMed. https://pubmed.ncbi.nlm.nih.gov/32342927/.

[37] Long Q.X., Liu B.Z., Deng H.J., Wu G.C., Deng K., Chen Y.K., Liao P., Qiu J.F., Lin Y., Cai X.F. (2020). Antibody responses to SARS-CoV-2 in patients with COVID-19. Nat. Med..

[38] Wan Y., Shang J., Sun S., Tai W., Chen J., Geng Q., He L., Chen Y., Wu J., Shi Z. (2019). Molecular Mechanism for Antibody-Dependent Enhancement of Coronavirus Entry. J. Virol..

[39] Song E., Zhang C., Israelow B., Lu-Culligan A., Prado A.V., Skriabine S., Lu P., Weizman O.E., Liu F., Dai Y. (2021). Neuroinvasion of SARS-CoV-2 in human and mouse brain. J. Exp. Med..

[40] Buzhdygan T.P., DeOre B.J., Baldwin-Leclair A., Bullock T.A., McGary H.M., Khan J.A., Razmpour R., Hale J.F., Galie P.A., Potula R. (2020). The SARS-CoV-2 spike protein alters barrier function in 2D static and 3D microfluidic in-vitro models of the human blood–brain barrier. Neurobiol. Dis..

[41] Al Saiegh F., Ghosh R., Leibold A., Avery M.B., Schmidt R.F., Theofanis T., Mouchtouris N., Philipp L., Peiper S.C., Wang Z.X. (2020). Status of SARS-CoV-2 in cerebrospinal fluid of patients with COVID-19 and stroke. J. Neurol. Neurosurg. Psychiatry.

[42] Neumann B., Schmidbauer M.L., Dimitriadis K., Otto S., Knier B., Niesen W.D., Hosp J.A., Günther A., Lindemann S., Nagy G. (2020). Cerebrospinal fluid findings in COVID-19 patients with neurological symptoms. J. Neurol. Sci..

[43] Destras G., Bal A., Escuret V., Morfin F., Lina B., Josset L. (2020). Systematic SARS-CoV-2 screening in cerebrospinal fluid during the COVID-19 pandemic. Lancet Microbe.

[44] Domingues R.B., Mendes-Correa M.C., de Moura Leite F.B.V., Sabino E.C., Salarini D.Z., Claro I., Santos D.W., de Jesus J.G., Ferreira N.E., Romano C.M. (2020). First case of SARS-COV-2 sequencing in cerebrospinal fluid of a patient with suspected demyelinating disease. J. Neurol..

[45] Andriuta D., Roger P.A., Thibault W., Toublanc B., Sauzay C., Castelain S., Godefroy O., Brochot E. (2020). COVID-19 encephalopathy: Detection of antibodies against SARS-CoV-2 in CSF. J. Neurol..

[46] Franke C., Ferse C., Kreye J., Reincke S.M., Sanchez-Sendin E., Rocco A., Steinbrenner M., Angermair S., Treskatsch S., Zickler D. (2021). High frequency of cerebrospinal fluid autoantibodies in COVID-19 patients with neurological symptoms. Brain Behav. Immun..

[47] de Antonio R., Suárez G., Barriuso F., Pérez R. (2021). Para-infectious anti-GD2/GD3 IgM myelitis during the Covid-19 pandemic: Case report and literature review. Mult. Scler. Relat. Disord..

[48] Águila-Gordo D., Flores-Barragán J.M., Ferragut-Lloret F., Portela-Gutierrez J., LaRosa-Salas B., Porras-Leal L., Guzmán J.C.V. (2020). Acute myelitis and SARS-CoV-2 infection. A new etiology of myelitis?. J. Clin. Neurosci..

[49] Armangue T., Spatola M., Vlagea A., Mattozzi S., Cárceles-Cordon M., Martinez-Heras E., Llufriu S. (2018). Frequency, symptoms, risk factors, and outcomes of autoimmune encephalitis after herpes simplex encephalitis: A prospective observational study and retrospective analysis. Lancet Neurol..

[50] Prüss H. (2017). Postviral autoimmune encephalitis: Manifestations in children and adults. Curr. Opin. Neurol..

[51] Hoffman L.A., Vilensky J.A. (2017). Encephalitis lethargica: 100 years after the epidemic. Brain.

[52] Matschke J., Lütgehetmann M., Hagel C., Sperhake J.P., Schröder A.S., Edler C., Mushumba H., Fitzek A., Allweiss L., Dandri M. (2020). Neuropathology of patients with COVID-19 in Germany: A post-mortem case series. Lancet Neurol..

[53] Tuma R.L., Guedes B.F., Carra R., Iepsen B., Rodrigues J., Camelo-Filho A.E., Kubota G., Ferrari M., Studart-Neto A., Oku M.H. (2021). Clinical, cerebrospinal fluid, and neuroimaging findings in COVID-19 encephalopathy: A case series. Neurol. Sci..

[54] Farhadian S., Glick L.R., Vogels C.B.F., Thomas J., Chiarella J., Casanovas-Massana A., Zhou J., Odio C., Vijayakumar P., Geng B. (2020). Acute encephalopathy with elevated CSF inflammatory markers as the initial presentation of COVID-19. BMC Neurol..

[55] Espíndola O.M., Brandão C.O., Gomes Y.C.P., Siqueira M., Soares C.N., Lima M.A.S.D., Leite A.C.C.B., Torezani G., Araujo A.Q.C., Silva M.T.T. (2021). Cerebrospinal fluid findings in neurological diseases associated with COVID-19 and insights into mechanisms of disease development. Int. J. Infect. Dis..

[56] Mondal R., Ganguly U., Deb S., Shome G., Pramanik S., Bandyopadhyay D., Lahiri D. (2020). Meningoencephalitis associated with COVID-19: A systematic review. J. Neurovirol..

[57] Ye M., Ren Y., Lv T. (2020). Encephalitis as a clinical manifestation of COVID-19. Brain Behav. Immun..

[58] Panariello A., Bassetti R., Radice A., Rossotti R., Puoti M., Corradin M., Moreno M., Percudani M. (2020). Anti-NMDA receptor encephalitis in a psychiatric Covid-19 patient: A case report. Brain Behav. Immun..

[59] Monti G., Giovannini G., Marudi A., Bedin R., Melegari A., Simone A.M., Santangelo M., Pignatti A., Bertellini E., Trenti T. (2020). Anti-NMDA receptor encephalitis presenting as new onset refractory status epilepticus in COVID-19. Seizure.

[60] Sarma D., Bilello L. (2020). A Case Report of Acute Transverse Myelitis Following Novel Coronavirus Infection. Clin. Pract. Cases Emerg. Med..

[61] Immunopathogenesis of Acute Transverse Myelitis: Current Opinion in Neurology. https://journals.lww.com/co-neurology/Citation/2002/06000/Immunopathogenesis_of_acute_transverse_myelitis.19.aspx.

[62] Munz M., Wessendorf S., Koretsis G., Tewald F., Baegi R., Krämer S., Geissler M., Reinhard M. (2020). Acute transverse myelitis after COVID-19 pneumonia. J. Neurol..

[63] AlKetbi R., AlNuaimi D., AlMulla M., AlTalai N., Samir M., Kumar N., AlBastaki U. (2020). Acute myelitis as a neurological complication of Covid-19: A case report and MRI findings. Radiol Case Reports..

[64] Abu-Rumeileh S., Abdelhak A., Foschi M., Tumani H., Otto M. (2020). Guillain–Barré syndrome spectrum associated with COVID-19: An up-to-date systematic review of 73 cases. J. Neurol..

[65] Dufour C., Co T.-K., Liu A. (2021). GM1 Ganglioside Antibody and COVID-19 related Guillain Barre Syndrome—A Case Report, Systemic Review and Implication for Vaccine Development. Brain, Behav. Immun. Health.

[66] Willison H.J., Jacobs B.C., van Doorn P.A. (2016). Guillain-Barré syndrome. Lancet.

[67] Trujillo Gittermann L.M., Valenzuela Feris S.N., von Oetinger Giacoman A. (2020). Relation between COVID-19 and Guillain-Barré syndrome in adults. Systematic review. Neurologia.

[68] Hasan I., Saif-Ur-Rahman K.M., Hayat S., Papri N., Jahan I., Azam R., Ara G., Islam Z. (2020). Guillain-Barrésyndrome associated with SARS-CoV-2 infection: A systematic review and individual participant data meta-analysis. J. Peripher. Nerv. Syst..

[69] Llorente Ayuso L., Torres Rubio P., Beijinho do Rosário R.F., Giganto Arroyo M.L., Sierra-Hidalgo F. (2020). Bickerstaff encephalitis after COVID-19. J. Neurol..

[70] Khoo A., McLoughlin B., Cheema S., Weil R.S., Lambert C., Manji H., Zandi M.S., Morrow J.M. (2020). Postinfectious brainstem encephalitis associated with SARS-CoV-2. J. Neurol. Neurosurg. Psychiatry.

[71] Baker D., Amor S., Kang A.S., Schmierer K., Giovannoni G. (2020). The underpinning biology relating to multiple sclerosis disease modifying treatments during the COVID-19 pandemic. Mult. Scler. Relat. Disord..

[72] Salmi A., Ziola B., Hovi T., Reunanen M. (1982). Antibodies to coronaviruses OC43 and 229E in multiple sclerosis patients. Neurology.

[73] Comi G., Cook S., Giovannoni G., Rieckmann P., Sørensen P.S., Vermersch P., Galazka A., Nolting A., Hicking C., Dangond F. (2019). Effect of cladribine tablets on lymphocyte reduction and repopulation dynamics in patients with relapsing multiple sclerosis. Mult. Scler. Relat. Disord..

[74] Celius E.G. (2020). Normal antibody response after COVID-19 during treatment with cladribine. Mult. Scler. Relat. Disord..

[75] Preziosa P., Rocca M.A., Nozzolillo A., Moiola L., Filippi M. (2020). COVID-19 in cladribine-treated relapsing-remitting multiple sclerosis patients: A monocentric experience. J. Neurol..

[76] Gelibter S., Orrico M., Filippi M., Moiola L. (2021). COVID-19 with no antibody response in a multiple sclerosis patient treated with cladribine: Implication for vaccination program?. Mult. Scler. Relat. Disord..

[77] Bar-Or A., Calkwood J.C., Chognot C., Evershed J., Fox E.J., Herman A., Manfrini M., McNamara J., Robertson D.S., Stokmaier D. (2020). Effect of ocrelizumab on vaccine responses in patients with multiple sclerosis: The VELOCE study. Neurology..

[78] Lucchini M., Bianco A., Del Giacomo P., De Fino C., Nociti V., Mirabella M. (2020). Is serological response to SARS-CoV-2 preserved in MS patients on ocrelizumab treatment? A case report. Mult. Scler. Relat. Disord..

[79] Boyaka P.N. (2017). Inducing Mucosal IgA: A Challenge for Vaccine Adjuvants and Delivery Systems. J. Immunol..

[80] Maillart E., Papeix C., Lubetzki C., Roux T., Pourcher V., Louapre C. (2020). Beyond COVID-19: DO MS/NMO-SD patients treated with anti-CD20 therapies develop SARS-CoV2 antibodies?. Mult. Scler. Relat. Disord..

[81] Thornton J.R., Harel A. (2020). Negative SARS-CoV-2 antibody testing following COVID-19 infection in Two MS patients treated with ocrelizumab. Mult. Scler. Relat. Disord..

[82] Conte W.L. (2020). Attenuation of antibody response to SARS-CoV-2 in a patient on ocrelizumab with hypogammaglobulinemia. Mult. Scler. Relat. Disord..

[83] Zabalza A., Cárdenas-Robledo S., Tagliani P., Arrambide G., Otero-Romero S., Carbonell-Mirabent P., Rodriguez-Barranco M., Rodríguez-Acevedo B., Restrepo Vera J.L., Resina-Salles M. (2020). COVID-19 in multiple sclerosis patients: Susceptibility, severity risk factors and serological response. Eur. J. Neurol..

[84] Milo R. (2020). Ofatumumab—A Potential Subcutaneous B-cell Therapy for Relapsing Multiple Sclerosis. Eur. Neurol. Rev..

[85] Flores-Gonzalez R.E., Hernandez J., Tornes L., Rammohan K., Delgado S. (2021). Development of SARS-CoV-2 IgM and IgG antibodies in a relapsing multiple sclerosis patient on ofatumumab. Mult. Scler. Relat. Disord..

[86] Chan A., De Seze J., Comabella M. (2016). Teriflunomide in Patients with Relapsing-Remitting Forms of Multiple Sclerosis. CNS Drugs.

[87] Luca B., Tommaso G., Bavaro D.F., Laura M., Annalisa S., Gioacchino A., Damiano P., Maria T., Pietro I. (2020). Seroconversion and indolent course of COVID-19 in patients with multiple sclerosis treated with fingolimod and teriflunomide. J. Neurol. Sci..

[88] Bar-Or A., Freedman M.S., Kremenchutzky M., Menguy-Vacheron F., Bauer D., Jodl S., Truffinet P., Benamor M., Chambers S., O’Connor P.W. (2013). Teriflunomide effect on immune response to influenza vaccine in patients with multiple sclerosis. Neurology.

[89] Okba N.M.A., Muller M.A., Li W. (2020). SARS-CoV-2 specific antibody responses in COVID-19 patients. medRxiv.

[90] Iadecola C., Anrather J., Kamel H. (2020). Effects of COVID-19 on the Nervous System. Cell.

[91] Berger J.R., Brandstadter R., Bar-Or A. (2020). COVID-19 and MS disease-modifying therapies. Neurol. Neuroimmunol. Neuroinflamm..

[92] Louapre C., Collongues N., Stankoff B., Giannesini C., Papeix C., Bensa C., Deschamps R., Créange A., Wahab A., Pelletier J. (2020). Clinical Characteristics and Outcomes in Patients with Coronavirus Disease 2019 and Multiple Sclerosis. JAMA Neurol..

[93] Baker D., Roberts C.A.K., Pryce G., Kang A.S., Marta M., Reyes S., Schmierer K., Giovannoni G., Amor S. (2020). COVID-19 vaccine-readiness for anti-CD20-depleting therapy in autoimmune diseases. Clin. Exp Immunol..

